# Resistin Induces Hypertension and Insulin Resistance in Mice via a TLR4-Dependent Pathway

**DOI:** 10.1038/srep22193

**Published:** 2016-02-26

**Authors:** Yun Jiang, Linfang Lu, Youtao Hu, Qiang Li, Chaoqiang An, Xiaolan Yu, Le Shu, Ao Chen, Congcong Niu, Lei Zhou, Zaiqing Yang

**Affiliations:** 1 College of Life Science and Technology, Huazhong Agricultural University, Wuhan, China; 2 State Key Laboratory for Conservation and Utilization of Subtropical Agro-bioresources, College of Animal Science and Technology, Guangxi University, Nanning, China; 3 Clinical Laboratory, the Third Hospital of Wuhan, Wuhan, China

## Abstract

Resistin, an adipokine involved in insulin resistance (IR) and diabetes, has recently been reported to play a role in cardiovascular events. However, its effect on blood pressure (BP) and the underlying mechanisms remain unclear. In the present study, we showed that resistin induces hypertension and IR in wild type (WT) mice, but not in *tlr4*^*−/−*^ mice. Resistin upregulated angiotensinogen (Agt) expression in WT mice, whereas it had no effect on *tlr4*^*−/−*^ mice, or in mice treated with the angiotensin-converting enzyme inhibitor perindopril. Real-time PCR and chromatin immunoprecipitation further confirmed that resistin activates the renin-angiotensin system (RAS) via the TLR4/P65/Agt pathway. This finding suggested an essential role of resistin in linking IR and hypertension, which may offer a novel target in clinic on the study of the association between diabetes and hypertension.

Hypertension, one of the independent risk factors for cardiovascular disease (CVD), affects approximately 70% of patients with diabetes, and the risk of CVD in diabetic patients is three times higher than that in healthy individuals^1^,^2^. Clinical studies suggest that insulin resistance (IR) and hyperinsulinemia, which often occur in patients with type 2 diabetes, are responsible for diabetes-associated hypertension^3^,^4^.

Resistin was first identified as an adipokine with a critical role in IR^5^,^6^,^7^. High plasma resistin levels have been reported in patients with CVD, indicating that increased resistin may be associated with both diabetes and CVD^8^. Accumulating evidence has provided insight into the function of resistin, and has implicated resistin in atherosclerosis, insulin-evoked vasodilation, and endothelial dysfunction, which are complications typically associated with hypertension^8^,^9^,^10^. These data suggest that resistin could be involved in the regulation of blood pressure (BP).

Toll-like receptor 4 (TLR4) is a putative resistin receptor that has been proposed to contribute to resistin-induced inflammation and IR^11^,^12^. However, whether resistin and TLR4 play a role in hypertension is largely unknown. In the present study, we investigated the effect of resistin on BP and IR in mice and elucidated the underlying mechanisms.

## Results

### Resistin induced hypertension and insulin resistance in wild-type mice

**Figure 1:**
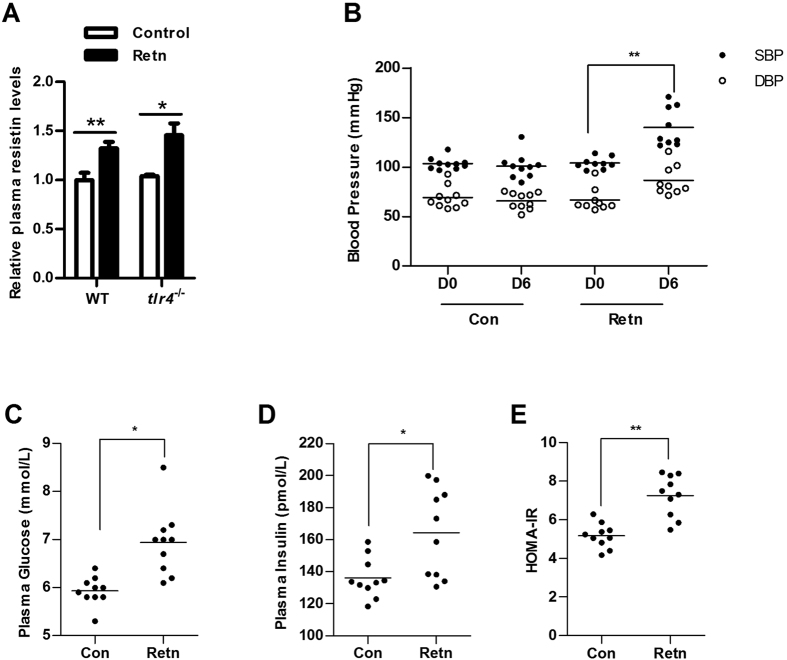
Effect of resistin on hypertension and insulin resistance in mice. (**A**) Plasma resistin levels were measured in control and resistin-treated (retn) mice after 6 days PBS or resistin treatment; (**B**) Blood pressure (BP) in wild-type (WT) mice. BP was measured before resistin treatment (day 0, D0) and after 6 days of resistin treatment (day 6, D6); (**C**) Plasma glucose levels; (**D**) Plasma insulin levels; (**E**) HOMA-IR was calculated using the following formula: fasting glucose (mmol/L) × fasting insulin (mU/ml)/22.5. Retn group was injected with 400 ng/day resistin, while the control group was injected with PBS. Injections were performed once per day for 6 consecutive days. Data are presented as mean ± sd (n = 9–10). *P < 0.05, **P < 0.01.

To examine the effect of resistin on hypertension, BP was measured in WT mice treated with resistin (Retn) or PBS (Con) using the tail-cuff method. Plasma resistin levels were higher in resistin-treated group than control group, indicating our resistin treatment is successful (Fig. 1A). Both systolic BP (SBP) and diastolic BP (DBP) were markedly higher in WT mice treated with resistin for 6 days [Fig. 1B, SBP: 104 ± 6 (Retn, D0), 140 ± 20 (Retn, D6) mmHg; DBP: 67 ± 12 (Retn, D0), 87 ± 15 (Retn, D6) mmHg], whereas no differences were observed in WT mice treated with PBS [Fig. 1B, SBP: 103 ± 6 (Con, D0), 101 ± 13 (Con, D6) mmHg; DBP: 69 ± 11 (Con, D0), 66 ± 8 (Con, D6) mmHg], indicating that resistin caused hypertension in WT mice. Resistin treatment increased plasma glucose and insulin levels, and IR as determined by the homeostasis model assessment (HOMA-IR) (Fig. 1C–E), indicating that resistin induced IR in WT mice.

### The induction of hypertension and IR by resistin is abrogated in *tlr4* ^*−/−*^ mice

**Figure 2:**
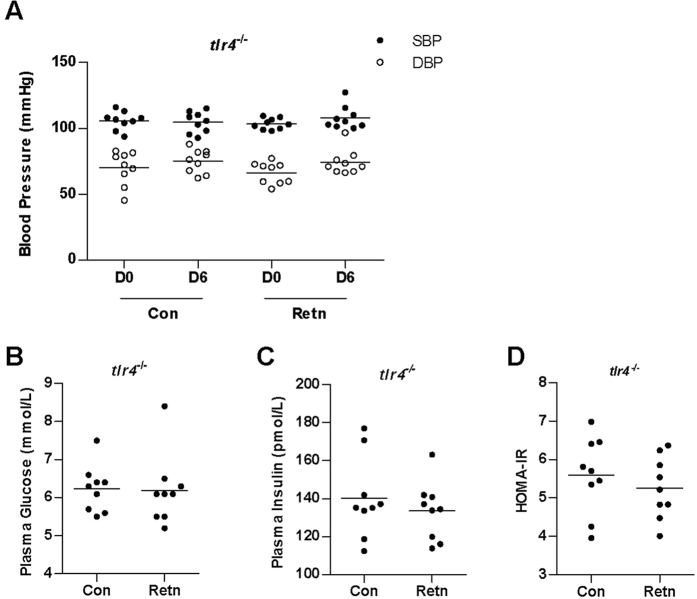
Effect of resistin on hypertension and IR in *tlr4*^*−/−*^ mice. (**A**) BP in *tlr4*^*−/−*^ mice. BP was measured before resistin treatment (day 0, D0) and after 6 days of resistin treatment (day 6, D6); (**B**) Plasma glucose levels; (**C**) Plasma insulin levels; (**D**) HOMA-IR was calculated using the following formula: fasting glucose (mmol/L) × fasting insulin (mU/ml)/22.5. Retn group was injected with 400 ng/day resistin, while the control group was injected with PBS. Injections were performed once per day for 6 consecutive days. Data are presented as mean ± sd (n = 9). *P < 0.05, **P < 0.01.

TLR4 is a putative resistin receptor; therefore, TLR4 knockout (*tlr4*^*−/−*^) mice were used to determine whether resistin induces hypertension and IR through TLR4. Plasma resistin levels were higher in resistin-treated group than control group, indicating our resistin treatment is successful (Fig. 1A). No significant differences in BP were observed between WT and *tlr4*^*−/−*^ mice treated with PBS [Fig. 1B, SBP in WT: 103 ± 6 (Con, D0), 101 ± 13 (Con, D6) mmHg; DBP in WT: 69 ± 11 (Con, D0), 66 ± 8 (Con, D6) mmHg; Fig. 2A, SBP in *tlr4*^*−/−*^: 106 ± 7 (Con, D0), 105 ± 8 (Con, D6) mmHg; DBP in *tlr4*^*−/−*^: 70 ± 13 (Con, D0), 75 ± 9 (Con, D6) mmHg], indicating that TLR4 deficiency had no effect on BP under normal conditions. However, BP values in resistin-treated *tlr4*^*−/−*^ mice [Fig. 2A, SBP in resistin-treated *tlr4*^*−/−*^: 103 ± 4 (Retn, D0), 108 ± 9 (Retn, D6) mmHg; DBP in resistin-treated *tlr4*^*−/−*^: 66 ± 8 (Retn, D0), 74 ± 9 (Retn, D6) mmHg] were similar to those observed in PBS-treated *tlr4*^*−/−*^ mice [Fig. 2A, SBP in PBS-treated *tlr4*^*−/−*^: 106 ± 7 (Con, D0), 105 ± 8 (Con, D6) mmHg; DBP in PBS-treated *tlr4*^*−/−*^: 70 ± 13 (Con, D0), 75 ± 9 (Con, D6) mmHg], indicating that resistin had no effect on BP in mice lacking TLR4. Moreover, resistin did not affect plasma glucose (Fig. 2B) and insulin (Fig. 2C) levels in *tlr4*^*−/−*^ mice, and no differences in HOMA-IR were observed (Fig. 2D). Taken together, these data demonstrate that the action of resistin on hypertension and IR is mediated by TLR4.

### Resistin activates the renin-angiotensin system by upregulating Agt expression

**Figure 3:**
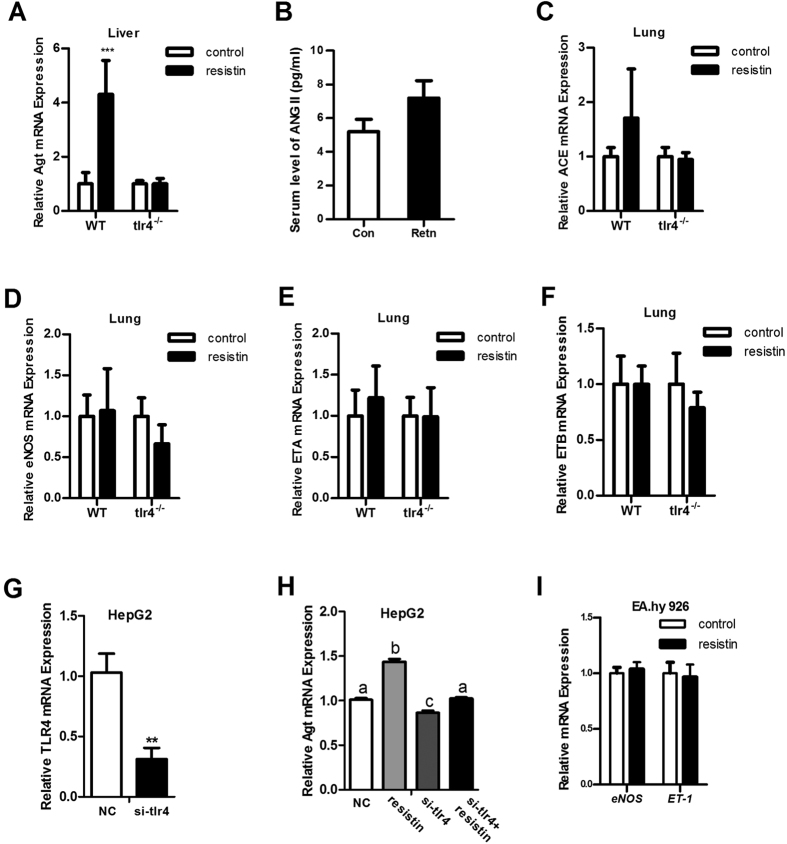
mRNA and plasma levels of BP-regulatory genes. mRNA and plasma levels of BP-regulatory genes were measured in WT mice and *tlr4*^*−/−*^ mice. (**A**) Angiotensinogen (Agt) was detected in the liver; (**B**) plasma ANG II levels; (**C**) ACE, (**D**) eNOS, (**E**) ETA, and (**F**) ETB were detected in the lung; (**G**) Inhibition efficiency of *tlr4* by siRNA, NC group was transfected with scrambled siRNA; (**H**) Inhibiting *tlr4* blocked the stimulation effect of resistin on Agt expression, NC and resistin groups were transfected with scrambled siRNA; (**I**) eNOS and ET-1 were measured in EA.hy 926 cells. Data are presented as mean ± sd. (for studies *in vivo*, n = 3–6; for studies *in vitro*, 3 independent experiments are performed) **P < 0.01, ***P < 0.001.

To investigate the mechanistic basis for resistin-induced hypertension, the mRNA levels of certain BP-regulatory genes were measured. Resistin significantly upregulated Agt mRNA expression in the liver of WT mice, whereas it had no effect in *tlr4*^*−/−*^ mice (Fig. 3A). Plasma ANG II level, the active form of Agt, was not significantly different, although a trend toward an increase in resistin-treated mice was observed (Fig. 3B). Similar mRNA levels of angiotensin-converting-enzyme (ACE), endothelial nitric oxide synthase (eNOS), and endothelin receptors A (ETA) and B (ETB) were detected in the lungs of WT and *tlr4*^*−/−*^ mice (Fig. 3C–F). In addition, renin (Ren), angiotensin-converting-enzyme 2 (ACE2) and angiotensin II receptor type 1a (Agtr1a) levels were not affected by resistin in WT mice (data not shown). Similar results were obtained in *in vitro* studies. After 24 h of resistin treatment, Agt mRNA was significantly upregulated in HepG2 cells (Fig. 3H), whereas endothelin-1 (ET-1) and eNOS levels remained constant in EA.hy 926 endothelial cells (Fig. 3I). Subsequently, siRNA was used to inhibit *tlr4* expression. After 24 h, Agt expression was detected in HepG2 cells. The data showed siRNA dramatically suppressed *tlr4* expression and this effect blocked the stimulation effect of resistin on Agt expression (Fig. 3G–H). These data indicated that resistin specifically stimulates Agt expression in the liver and this effect is TLR4-dependent.

**Figure 4:**
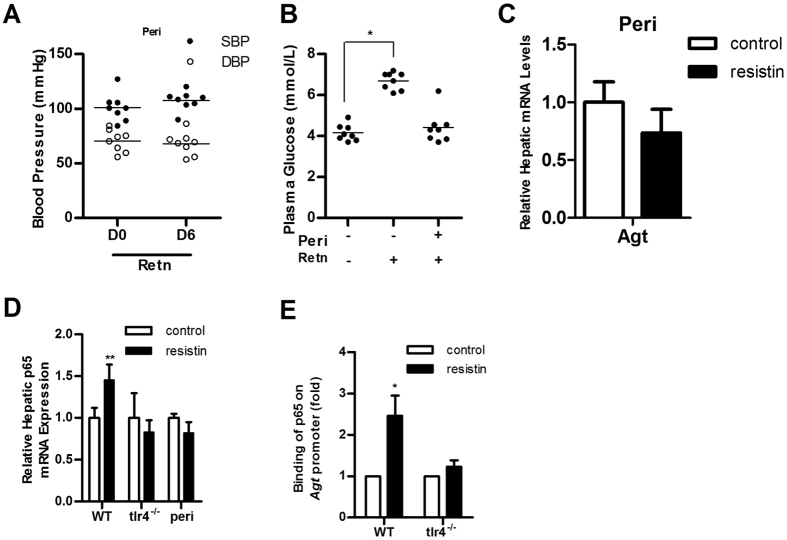
Perindopril blocks the action of resistin. (**A**) BP in wild-type mice pre-treated with perindopril (Peri). BP was measured before resistin treatment (day 0, D0) and after 6 days of resistin treatment (day 6, D6); (**B**) Plasma glucose levels in mice exposed to different treatments; (**C**) Agt and (**D**) p65 expression in mice exposed to different treatments and in different mouse lines; (**E**) Binding of p65 to the Agt promoter was determined by chromatin immunoprecipitation. Retn group was injected with 400 ng/day resistin, while the control group was injected with PBS. Perindopril (5 mg/kg/day) was administered orally for 7 days (animals were treated as described in Materials and Methods). Data are presented as mean ± sd (n = 8). *P < 0.05, **P < 0.01.

As a precursor of Angiotensin I (ANG I), Agt is crucial to the renin-angiotensin system (RAS), which is known as a classical blood pressure regulation system. Therefore, the signal transduction pathway of resistin was further examined by blocking the RAS using the ACE inhibitor perindopril (peri). Resistin had no effect on SBP and DBP in WT mice pre-treated with perindopril (Fig. 4A), and plasma glucose levels were also unchanged (Fig. 4B). Moreover, resistin-induced upregulation of Agt was inhibited by pre-treatment with perindopril (Fig. 4C). These findings indicate that resistin-induced hypertension is dependent on the activation of the RAS.

### Resistin induces Agt expression by activating the TLR4/P65 pathway

In our previous study, resistin upregulated p65 mRNA and protein expression^13^. Consistently, in the present study, hepatic p65 expression was upregulated in WT mice treated with resistin, but not in *tlr4*^*−/−*^ or perindopril-treated mice (Fig. 4D). Deb *et al.* identified a p65 binding site in the mouse Agt promoter, implying that p65 can directly activate Agt transcription^14^. To determine whether p65 is involved in resistin-induced Agt upregulation, we used ChIP to quantify the occupancy of p65 on the Agt promoter. Our data showed that resistin treatment increased binding of p65 to the Agt promoter in WT mice, but this effect was abolished in *tlr4*^*−/−*^ mice (Fig. 4E). These results support that resistin activates the RAS through the TLR4/P65/Agt pathway.

## Discussion

Epidemiological studies have demonstrated that patients with diabetes are more likely to develop hypertension, which increases their risk for CVD^2^. Moreover, as a major pathological feature of type 2 diabetes, IR has often been reported occur in the people with essential hypertension, suggesting these two pathological conditions may develop through a shared pathway^3^,^15^. Resistin has been suggested to induce IR and is associated with the development of CVD^8^. However, the role of resistin in the regulation of BP and the development of diabetes and hypertension remains unclear. In the present study, we investigated the effect of resistin on BP and IR. Our data showed that resistin induces both IR and hypertension in mice and these effects are TLR4-dependent.

Studies in rodents and humans have suggested that resistin promotes IR^6^,^7^, although contradictory findings have also been reported^16^,^17^. In the present study, plasma glucose and insulin levels, as well as HOMA-IR, were elevated in WT mice after resistin treatment (Fig. 1C–E), confirming that resistin induces IR. To investigate the role of resistin in the development of hypertension, WT and *tlr4*^*−/−*^ mice were treated with resistin for 6 days. Both SBP and DBP increased in WT mice, whereas no changes in BP were observed in *tlr4*^*−/−*^ mice, indicating that resistin likely regulates BP through TLR4-mediated signaling. In addition, resistin had no effect on plasma glucose and insulin levels or HOMA-IR in *tlr4*^*−/−*^ mice (Fig. 2B–D), suggesting that resistin-induced IR is also TLR4-dependent.

Previous studies have implicated resistin in the regulation of certain vasoconstrictors and vasodilators^9^,^18^,^19^,^20^. Here, we linked resistin with RAS, which is a hormone system involved in BP regulation. As a precursor of ANG II, Agt is released from the liver and converted into ANG I through the action of renin. Then, ANG I is converted to ANG II (a major RAS effector) by ACE^21^,^22^. An increase in Agt expression could cause an increase in ANG II and activation of RAS, leading to an elevation in BP. In the present study, Agt expression in the mouse liver was significantly upregulated by resistin treatment (Fig. 3A). Pre-treatment of mice with the ACE inhibitor perindopril abolished resistin-induced hypertension and IR, suggesting that the action of resistin is inhibited when the RAS is blocked. There is considerable evidence demonstrating the activation of the NF-κB pathway by resistin^23^,^24^,^25^ and resistin was previously found to enhance p65 mRNA and protein expression in the liver^13^. As a major transcription factor in the NF-κB pathway, p65 (Rel A) can directly bind to the promoter of Agt and thereby upregulate Agt expression^14^. Therefore, we presumed that resistin-induced upregulation of Agt expression could be mediated by p65. Quantification of mRNA levels indicated that resistin upregulated the expression of p65 in WT mice, but not in *tlr4*^*−/−*^ or perindopril-treated mice (Fig. 4D), corresponding to the upregulation of Agt expression. ChIP data showed that resistin promoted the binding of p65 to the Agt promoter in WT but not *tlr4*^*−/−*^ mice (Fig. 4E), indicating that resistin upregulates Agt expression via the TLR4/p65 pathway.

Unlike mice, in human, resistin is produced mainly in macrophages rather than in adipocytes^26^, therefore, whether its effect on rodents is translatable to human is not fully confirmed^27^,^28^. However, an elevated resistin level has been observed in the patient with IR or type 2 diabetes in many clinical studies and in the patients with essential hypertension according to some studies^29^,^30^. This also fits to the finding in our mouse model on the importance of resistin linking IR and hypertension. However, dual to the complexity in human epidemiology, it’s difficult to address whether resistin is an active player in the development of those pathological conditions especially hypertension in diabetes patients. Therefore, some literatures suggest effect of resistin on diabetic hypertension may be through elevated ET-1 or reduced eNOS^31^,^32^ which we didn’t observed in our mouse model (Fig. 3D) as well as *in vitro* study (Fig 3F). Here, we have found the role of resistin in the activation of RAS in our mouse model which offered another possible explanation for the association of resistin with diabetes and hypertension.

Taken together, our data indicate that resistin induces IR and hypertension in mice via a mechanism dependent on TLR4 and RAS, suggesting that resistin is a crucial factor linking diabetes and hypertension. The results of the present study support the existence of a common pathway underlying the development of diabetes and hypertension, and provide a novel animal model to further investigate this issue.

## Methods

### Animals

Male C57BL/10 (wild-type, WT) and C57BL/10ScN (TLR4 gene deleted type, *tlr4*^*−/−*^) mice (7–8 weeks old) received daily intravenous injections of PBS or 400 ng/day of recombinant mouse resistin (R&D Systems, Minneapolis, MN, USA) for six consecutive days. For the renin-angiotensin system (RAS) blocking experiment, mice were pre-treated one day prior to the start of the injection regimen with the angiotensin-converting enzyme (ACE) inhibitor perindopril (5 mg/kg; mBbio, Nanjing, China) by oral administration, and perindopril was administered 30 min before each injection. BP was measured with a tail-cuff system (BP-98A; Gene & I Co. Ltd, Beijing, China). Retro-orbital blood was collected after 12 h of food withdrawal and mice were directly sacrificed afterwards. Plasma glucose levels were determined by the glucose oxidase method using a glucose determination kit (Applygen Technologies Inc, Beijing, China). Insulin and resistin levels were measured with an insulin ELISA kit (Xinqidi Biological Technology Co. Ltd, Wuhan, China).All procedures were performed in strict accordance with the recommendations in the Guide for the Care and Use of Laboratory Animals of Hubei Province. All animal experiments were performed under approval of the Institutional Animal Care and Use Committee (IACUC) (protocol: 00116272) of Wuhan University. The protocol was approved by the Committee on the Ethics of Animal Experiments of Wuhan University (Permit Number: 2013030).

### Cell Culture and Treatment

Agt gene expression was assessed in HepG2 cells, and nitric oxide synthase (NOS) and endothelin-1 (ET-1) were assessed in EA.hy 926 endothelial cells. Cells were grown in Dulbecco’s modified Eagle’s medium (DMEM) supplemented with 10% fetal bovine serum and cultured under 5% CO_2_ at 37 °C. The control group was cultured without recombinant resistin, whereas the treatment group was cultured with recombinant resistin (50 ng/mL). Cells were collected 24 h after treatment for RNA isolation.

### RNA extractNion and cDNA synthesis

Total RNA was isolated with TRIzol (Takara, Dalian, China) according to the manufacturer’s protocol. All RNA samples were treated with DNase I to remove genomic DNA prior to cDNA synthesis. RNA concentration was determined using a SMA4000 UV-Vis Spectrophotometer (Merinton, Beijing, China). Total RNA was reverse-transcribed using a cDNA Synthesis kit (Takara, Dalian, China).

### Real-time PCR

For determination of gene expression in mice, the β-actin gene was used as the internal control; for gene expression in HepG2 cells and EA.hy 926 endothelial cells, the GAPDH gene was used as the internal control. Primer sequences and accession numbers for each gene are listed in Supplemental Table S1.

### Chromatin immunoprecipitation (ChIP)

ChIP was performed using the SimpleChIP Plus Enzymatic Chromatin IP Kit (Magnetic Beads) (Cell Signaling Technology, Inc., Beverly, MA, USA) following the manufacturer’s instructions. For each immunoprecipitation reaction, 5 μg of mouse P65 antibody (Santa Cruz, CA, USA) was used for every 5 μg of pre-cleared chromatin. Recovered DNA was quantified by real-time PCR. The primer sequences for amplifying P65 binding sites on the mouse Agt promoter have been previously described^14^.

### Statistical analysis

Results are presented as the mean ± sd. Statistical analyses were performed using an unpaired two-tailed *t*-test, or ANOVA for more than two groups. The correlation was estimated by partial correlation analysis after adjustment for gender, age, and body weight. *P* < 0.05 was considered statistically significant.

## Additional Information

**How to cite this article**: Jiang, Y. *et al.* Resistin Induces Hypertension and Insulin Resistance in Mice via a TLR4-Dependent Pathway. *Sci. Rep.* **6**, 22193; doi: 10.1038/srep22193 (2016).

## Supplementary Material

Supplementary Table

## References

[b1] Lago R. M., Singh P. P. & Nesto R. W. Diabetes and hypertension. Nature Clinical Practice Endocrinology & Metabolism. 3, 667–667 (2007). 10.1038/ncpendmet0638 17893686

[b2] Sowers J. R., Epstein M. & Frohlich E. D. Diabetes, hypertension, and cardiovascular disease: an update. Hypertension. 37, 1053–9 (2001). 11304502 10.1161/01.hyp.37.4.1053

[b3] DeFronzo R. A. & Ferrannini E. Insulin resistance. A multifaceted syndrome responsible for NIDDM, obesity, hypertension, dyslipidemia, and atherosclerotic cardiovascular disease. Diabetes Care. 14, 173–94 (1991). 2044434 10.2337/diacare.14.3.173

[b4] Groop L. *et al.* Insulin resistance, hypertension and microalbuminuria in patients with type 2 (non-insulin-dependent) diabetes mellitus. Diabetologia. 36, 642–647 (1993). 8359582 10.1007/BF00404074

[b5] Koerner A., Kratzsch J. & Kiess W. Adipocytokines: leptin–the classical, resistin–the controversical, adiponectin-the promising, and more to come. Best Pract Res Clin Endocrinol Metab. 19, 525–46 (2005). 16311215 10.1016/j.beem.2005.07.008

[b6] Muse E. D. *et al.* Role of resistin in diet-induced hepatic insulin resistance. J Clin Invest. 114, 232–9 (2004). 15254590 10.1172/JCI21270 PMC449748

[b7] Steppan C. M. *et al.* The hormone resistin links obesity to diabetes. Nature. 409, 307–12 (2001). 11201732 10.1038/35053000

[b8] Jamaluddin M. S., Weakley S. M., Yao Q. & Chen C. Resistin: functional roles and therapeutic considerations for cardiovascular disease. Br J Pharmacol. 165, 622–632 (2012). 21545576 10.1111/j.1476-5381.2011.01369.x PMC3315035

[b9] Gentile M. T. *et al.* Resistin impairs insulin-evoked vasodilation. Diabetes. 57, 577–83 (2008). 18065520 10.2337/db07-0557

[b10] Calabro P., Samudio I., Willerson J. T. & Yeh E. T. Resistin promotes smooth muscle cell proliferation through activation of extracellular signal–regulated kinase 1/2 and phosphatidylinositol 3-kinase pathways. Circulation. 110, 3335–3340 (2004). 15545519 10.1161/01.CIR.0000147825.97879.E7

[b11] Benomar Y. *et al.* Central resistin overexposure induces insulin resistance through Toll-like receptor 4. Diabetes. 62, 102–114 (2013). 22961082 10.2337/db12-0237 PMC3526022

[b12] Tarkowski A., Bjersing J., Shestakov A. & Bokarewa M. I. Resistin competes with lipopolysaccharide for binding to toll-like receptor 4. J Cell Mol Med. 14, 1419–31 (2010). 19754671 10.1111/j.1582-4934.2009.00899.x PMC3829009

[b13] Zhou L. *et al.* Resistin reduces mitochondria and induces hepatic steatosis in mice by the protein kinase C/protein kinase G/p65/PPAR gamma coactivator 1 alpha pathway. Hepatology. 57, 1384–93 (2013). 23174781 10.1002/hep.26167

[b14] Deb D. K. *et al.* 1, 25-Dihydroxyvitamin D3 suppresses high glucose-induced angiotensinogen expression in kidney cells by blocking the NF-κB pathway. American Journal of Physiology-Renal Physiology. 296, F1212–F1218 (2009). 19193728 10.1152/ajprenal.00002.2009 PMC2681355

[b15] Sowers J. R. Insulin resistance and hypertension. American Journal of Physiology-Heart and Circulatory Physiology. 286, H1597–H1602 (2004). 15072967 10.1152/ajpheart.00026.2004

[b16] Janke J., Engeli S., Gorzelniak K., Luft F. C. & Sharma A. M. Resistin gene expression in human adipocytes is not related to insulin resistance. Obesity research. 10, 1–5 (2002). 11786595 10.1038/oby.2002.1

[b17] Silha J. V. *et al.* Plasma resistin, adiponectin and leptin levels in lean and obese subjects: correlations with insulin resistance. European Journal of Endocrinology. 149, 331–335 (2003). 14514348 10.1530/eje.0.1490331

[b18] Verma S. *et al.* Resistin promotes endothelial cell activation: further evidence of adipokine-endothelial interaction. Circulation. 108, 736–40 (2003). 12874180 10.1161/01.CIR.0000084503.91330.49

[b19] Shen Y. H. *et al.* Up-regulation of PTEN (phosphatase and tensin homolog deleted on chromosome ten) mediates p38 MAPK stress signal-induced inhibition of insulin signaling A cross-talk between stress signaling and insulin signaling in resistin-treated human endothelial cells. Journal of Biological Chemistry. 281, 7727–7736 (2006). 16418168 10.1074/jbc.M511105200

[b20] Chen C. *et al.* Resistin decreases expression of endothelial nitric oxide synthase through oxidative stress in human coronary artery endothelial cells. American Journal of Physiology - Heart and Circulatory Physiology. 299, H193–H201 (2010). 20435848 10.1152/ajpheart.00431.2009 PMC2904138

[b21] Herichova I. & Szantoova K. Renin-angiotensin system: upgrade of recent knowledge and perspectives. Endocr Regul. 47, 39–52 (2013). 23363256 10.4149/endo_2013_01_39

[b22] Jeunemaitre X. *et al.* Molecular basis of human hypertension: role of angiotensinogen. Cell. 71, 169–80 (1992). 1394429 10.1016/0092-8674(92)90275-h

[b23] Bertolani C. *et al.* Resistin as an intrahepatic cytokine: overexpression during chronic injury and induction of proinflammatory actions in hepatic stellate cells. Am J Pathol. 169, 2042–53 (2006). 17148667 10.2353/ajpath.2006.060081 PMC1762467

[b24] Bokarewa M., Nagaev I., Dahlberg L., Smith U. & Tarkowski A. Resistin, an adipokine with potent proinflammatory properties. J Immunol. 174, 5789–95 (2005). 15843582 10.4049/jimmunol.174.9.5789

[b25] Lehrke M. *et al.* An inflammatory cascade leading to hyperresistinemia in humans. PLoS Med. 1, e45 (2004). 15578112 10.1371/journal.pmed.0010045 PMC529430

[b26] Steppan C. M. & Lazar M. A. The current biology of resistin. J Intern Med. 255, 439–447 (2004). 15049878 10.1111/j.1365-2796.2004.01306.x

[b27] Heilbronn L. *et al.* Relationship between serum resistin concentrations and insulin resistance in nonobese, obese, and obese diabetic subjects. Journal of Clinical Endocrinology & Metabolism. 89, 1844–1848 (2004). 15070954 10.1210/jc.2003-031410

[b28] Kusminski C. M., McTernan P. G. & Kumar S. Role of resistin in obesity, insulin resistance and Type II diabetes. Clin Sci (Lond). 109, 243–56 (2005). 16104844 10.1042/CS20050078

[b29] Fang C. *et al.* Association of higher resistin levels with inflammatory activation and endothelial dysfunction in patients with essential hypertension. Chinese medical journal. 126, 646–649 (2013). 23422182

[b30] Schwartz D. R. & Lazar M. A. Human resistin: found in translation from mouse to man. Trends in Endocrinology & Metabolism. 22, 259–265 (2011). 21497511 10.1016/j.tem.2011.03.005 PMC3130099

[b31] Chen C. *et al.* Resistin decreases expression of endothelial nitric oxide synthase through oxidative stress in human coronary artery endothelial cells. American Journal of Physiology-Heart and Circulatory Physiology. 299, H193–H201 (2010). 20435848 10.1152/ajpheart.00431.2009 PMC2904138

[b32] Verma S. *et al.* Resistin promotes endothelial cell activation further evidence of adipokine-endothelial interaction. Circulation. 108, 736–740 (2003). 12874180 10.1161/01.CIR.0000084503.91330.49

