# Supplementary material for: Resistin Induces Hypertension and Insulin Resistance in Mice via a TLR4-Dependent Pathway
Source: Sci Rep. 2016 Feb 26;6:22193. doi: 10.1038/srep22193 (PMC4768137; doi:10.1038/srep22193)
Supplement: Supplementary Table [file srep22193-s1.doc]

**Title: Resistin Induces Hypertension and Insulin Resistance in Mice via a TLR4-Dependent Pathway**

**Authors:** Yun Jiang^1^, Linfang Lu^1^, Youtao Hu^3^, Qiang Li^2^, Chaoqiang An^1^, Xiaolan Yu^1^, Le Shu^1^, Ao Chen^1^, Congcong Niu^1^, Lei Zhou^2*^, Zaiqing Yang^1*^

**Supplemental Table S1**

**Table S1. Primers** for real-time RT-PCR

| **Gene name** | **Size (bp)** | **Forward and Reverse primer (5’-3’)** | **Accession number** |
| --- | --- | --- | --- |
| Homo GAPDH | 147 | F: TGGACCTGACCTGCCGTCTAG  R: AGTGGGTGTCGCTGTTGAAGTC | NM_002046 |
| Homo p65 | 146 | F:ATCCCATCTTTGACAATCGTGC  R: CGTGAAATACACCTCAATGTCCTC | NM_021975 |
| Homo Agt | 158 | F: CTGGCTTTCAACACCTAC  R: TTGTCCTGGATGTCACTC | NM_000029 |
| Homo eNOS | 354 | F: CCAGCTAGCCAAAGTCACCAT  R: GTCTCGGAGCCATACAGGATT | NM_000603 |
| Homo ET1 | 224 | F: CTTGGGAAAAAGTGTATTTATCAGC  R: GGAGGCTATGGCTTCAGACAG | NM_001168319 |
| Mus β-actin | 154 | F: GGCTGTATTCCCCTCCATCG  R: CCAGTTGGTAACAATGCCATGT | NM_007393 |
| Mus P65 | 156 | F: CCAGACACAGATGATCGCCAC  R: GACAGAAGTTGAGTTTCGGGTAGG | NM_009045 |
| Mus Agt | 189 | F: CAAATCTGAACAACATTGGTGACA  R: TGCCTGAGTCCTGCTCGTAGAT | NM_007428 |
| Mus eNOS | 148 | F: TGTCTGCGGCGATGTCACTA  R: TCCGAAAATGTCCTCGTGGTA | NM_008713 |
| Mus ETA | 114 | F: TCCTCTGTTGCTGTTGTCACCA  R: TGCTCCGTTCCGTGTTGTG | NM_010332 |
| Mus ETB | 156 | F: CTGCATCAATCCAATCGCTC  R: CGGAAGTTGTCATATCCGTGAT | NM_001136061 |
| Mus Agtr1a | 239 | F: ATAACTCACAGCAACCCTCCAAG  R: CACCACCAAGCTGTTTCCAAA | NM_177322 |
| Mus ACE | 315 | F: CCAAGTGTTGTTGAACGAGTACG  R: GGCATACAAGTGCCATTTGTGT | NM_207624 |
| Mus ACE2 | 119 | F: TACCTTCGCAGAGATCAAGCC  R: CAAGCAAATGGGCAGGGA | NM_001130513 |
| Mus Ren | 157 | F: CCTACACACTCAGCAGTACGGAC  R: TCAAACTCTGTATAGAACTTGCGGA | NM_031192 |
